# The Diketopyrrolopyrrole (DPP) Core as a Gel-Forming Material: Current Status and Untapped Potential

**DOI:** 10.3390/gels11020134

**Published:** 2025-02-13

**Authors:** Abelardo Sánchez-Oliva, Iván Torres-Moya

**Affiliations:** Department of Inorganic, Organic Chemistry and Biochemistry, Faculty of Chemical Science and Technologies, Instituto Regional de Investigación Científica Aplicada (IRICA), University of Castilla-La Mancha, 13005 Ciudad Real, Spain; abelardo.sanchez@uclm.es

**Keywords:** gels, diketopyrrolopyrrole (DPP)

## Abstract

The diketopyrrolopyrrole (DPP) core is widely recognized for its applications in organic electronics and photonics due to its exceptional electronic and optical properties. Recently, DPP-based materials have shown remarkable π–π stacking interactions and tunable self-assembly, making them promising candidates for gel formation. However, the development of DPP-based gels remains in its infancy, primarily hindered by challenges such as limited gelation efficiency, poor mechanical robustness, and sensitivity to environmental conditions. Overcoming these issues is crucial for unlocking their full potential in functional soft materials. This review compiles and analyzes existing studies on DPP-containing gel systems, highlighting their structural versatility, self-assembly mechanisms, and advantages over conventional gelators. By examining these works, we identify key strategies for DPP gel formation, evaluate their physicochemical performance, and discuss innovative approaches to address current limitations. Finally, we propose future research directions to advance the field and establish DPP-based gels as a robust platform for next-generation soft materials.

## 1. Introduction

A gel is a material typically composed of a network of solid particles, small molecules, or macromolecules dispersed within a liquid phase, where the solid phase predominates and defines the material’s structure, imparting a unique set of mechanical properties. Unlike conventional liquids or solids, gels exhibit characteristics of both states—retaining their shape like solids while displaying flowability under specific conditions [1,2,3]. The structure of a gel is primarily governed by the network it forms, in which interactions between components occur through physical or chemical bonds, creating a three-dimensional scaffold capable of retaining significant amounts of liquid without flowing freely. This distinctive architecture makes gels highly versatile materials, enabling their application across a wide range of fields, including sensors [4,5,6], actuators [7,8,9], tissue engineering [10,11], drug delivery systems [12,13,14], drug crystallization [14,15,16,17], 3D/4D printing [18,19], food science and related industries [20,21,22], energy storage [23,24], agriculture [25,26], cosmetics [27,28,29], and even advanced applications in photonics, electronics, and photovoltaics [30].

Gels are typically formed using gelators, which are compounds capable of self-assembling into a three-dimensional network that entraps a solvent within its structure. These gelators can be classified into two main categories based on molecular weight: polymers [31] and small molecules [32]. Polymeric gelators include both natural biopolymers, such as agar [33], alginate [34], cellulose [35], gelatin [36], carrageenan [37], or sodium hyaluronate [38], and synthetic polymers, such as poly(vinyl alcohol) (PVA) [39], poly(ethylene glycol) (PEG) [40], polyacrylamide (PAM) [41], poly(N-isopropylacrylamide) (PNIPAM) [42], or sodium polyacrylate (PAAS) [43]. These materials are widely used due to their ability to form stable networks and their high tunability under various gelation conditions, making them particularly suitable for biomedical and pharmaceutical applications. In contrast, small-molecule gelators are low-molecular-weight compounds that self-assemble into gels through non-covalent interactions, including hydrogen bonding, van der Waals forces, and π–π stacking [44,45]. Examples of such gelators include fatty acids [46], surfactants [47], and peptides [48], which offer distinct advantages, such as responsiveness to environmental stimuli (e.g., temperature, pH, or light), enabling their application in dynamic and stimuli-responsive systems [49,50,51].

A key feature of gels is their ability to undergo gelation, a process in which a liquid transitions into a semi-solid state. This transformation occurs when the material’s components—typically polymers, particles, or small molecules—self-assemble into a network structure that entraps liquid within its pores. The mechanical properties of the resulting gel, including stiffness, elasticity, and thermal stability, are largely determined by the solid particle concentration or degree of crosslinking, which refers to the extent of connectivity between polymer chains. These properties can be further tailored by modifying factors such as the concentration of the gelator, the choice of solvent or the addition of co-solvents, the structure of the macromolecular chains, the molecular weight, the addition of solid particles, or external stimuli such as temperature and pH [52,53]. Due to their inherent flexibility and adaptability, gels are classified as “soft materials”, making them highly suitable for a wide range of applications, as previously discussed.

**Figure 1:**
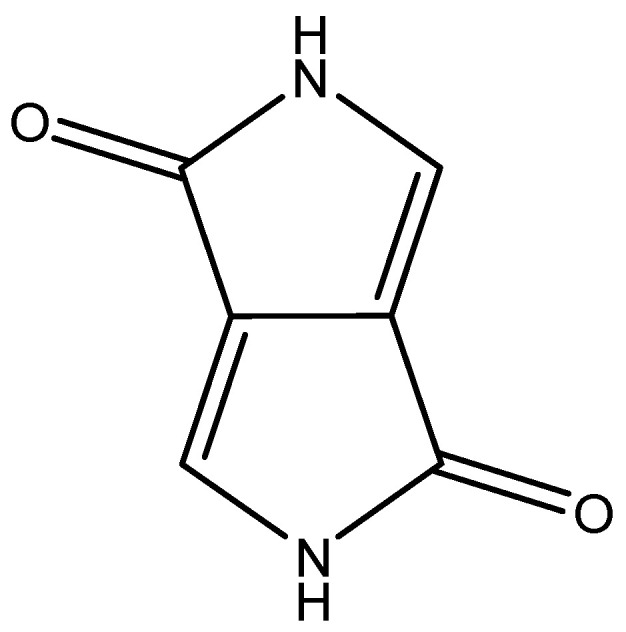
Chemical structure of Diketopyrrolopyrrole (DPP), object of analysis in this work.

In recent years, there has been growing interest in exploring novel, less conventional gelators that combine the desirable properties of both small molecules and polymers. One such compound is diketopyrrolopyrrole (DPP), a π-conjugated organic molecule (Figure 1) with remarkable characteristics, including strong π–π stacking interactions, high thermal stability, and tunable functionalization, extensively studied for its applications in photonics, electronics, and photovoltaics, including organic field-effect transistors [54,55], organic solar cells [56,57], organic light-emitting diodes [58,59], and optical waveguides [60,61]. The potential of DPP as a gel-forming material has only recently been explored, despite its inherent capacity for self-assembly and unique chemical structure with its high functionalization versatility, which offers significant advantages for gelation, making it a promising candidate for developing gels with tailored mechanical, thermal, and optical properties. To date, the few articles concerning the use of DPP as gels have been based on the functionalization of the backbone with hydrogen bonding groups such as amides or amino acids (phenylalanine (F), tyrosine (Y), and leucine (L)), as will be detailed in this review paper. These gels have shown applications in bio fields or, for example, in solar cells, demonstrating the wide variety of possibilities offered by this core. A correct functionalization of the DPP can mark the working direction of the obtained gels, as well as the obtaining of multifunctional gels. However, the limited number of studies investigating DPP-based gels highlights an untapped opportunity to further understand its gelation and optimize its properties through chemical modifications. This review examines the existing examples of DPP-based gels, the key factors influencing their formation, and their potential applications across various fields, aiming to emphasize the versatility of DPP as a gelator and to encourage further research in this promising area.

## 2. Results and Discussion

**Figure 2:**
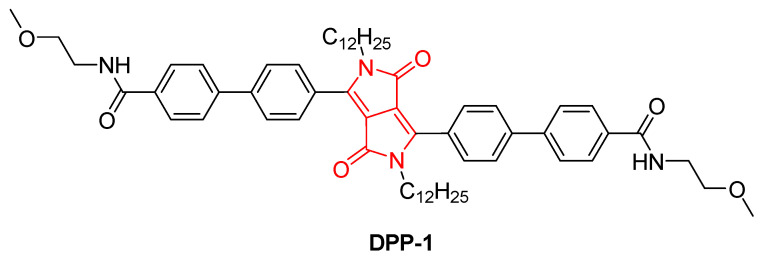
Chemical structure of **DPP-1**, described by Thool et al. (2016) [62].

To the best of our knowledge, the first reported example of DPP-based gels was described by Thool et al. in 2016 [62]. In that study, the authors introduced a novel thermoreversible organogel, **DPP-1** (Figure 2), which forms a one-dimensional supramolecular assembly for electronic applications, particularly in organic solar cells (OSCs).

**Figure 3:**
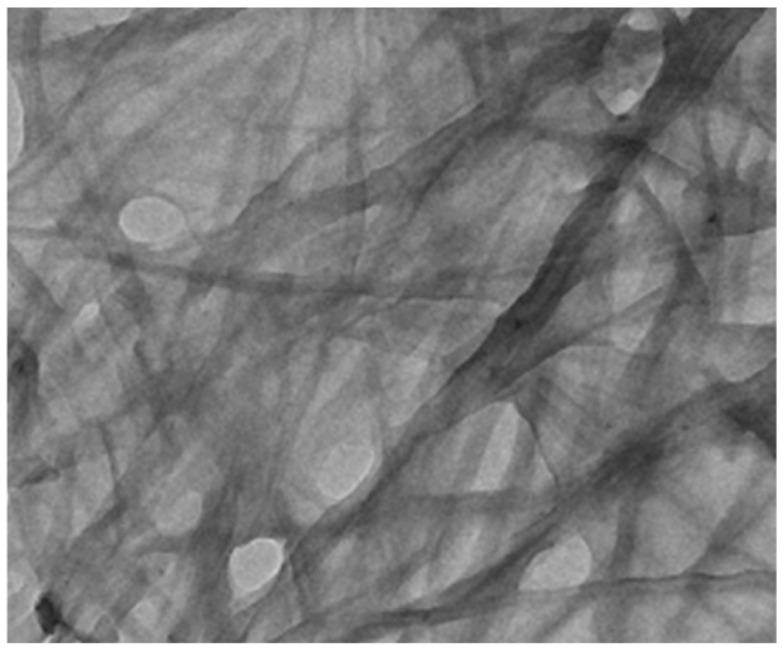
HRTEM images of **DPP-1** xerogel in chlorobenzene [62]. Image taken from reference [62]. Reprinted (adapted) with permission from (Langmuir **2016**, 32, 4346–4351). Copyright (2025). American Chemical Society.

**DPP-1** is characterized by its ability to form gels through π–π stacking between DPP molecules and hydrogen bonding interactions because of the amide groups of the peripheral chains. The study demonstrates that **DPP-1** exhibits a strong tendency to self-assemble into one-dimensional (1D) rod-like structures, as confirmed by atomic force microscopy (AFM) and high-resolution transmission electron microscopy (HRTEM) images (Figure 3). These 1D structures, which display a high aspect ratio (length-to-width ratio), are particularly desirable in organic electronics due to their exceptional charge transport and conductive properties.

**Figure 4:**
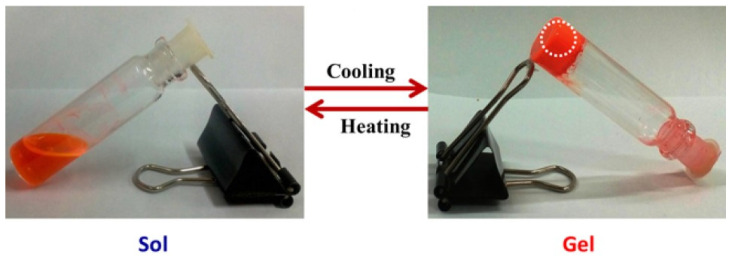
Thermoreversible capacity of **DPP-1** depending on temperature conditions [62]. Image taken from reference [62]. Reprinted (adapted) with permission from (Langmuir **2016**, 32, 4346–4351). Copyright (2025). American Chemical Society.

The gelation process of **DPP-1** was found to be thermoreversible, allowing the gel to form and reform in response to temperature variations. This thermoreversible behavior was achieved by mixing the gelator with different solvents (DMSO, DMF, ethyl acetate, chlorobenzene, acetone, and hexane) at room temperature, followed by heating and subsequent cooling to approximately 10 °C (Figure 4), leading to rapid gel formation within less than five minutes. This efficient and easily controllable process suggests that **DPP-1** holds significant potential for industrial and device applications requiring flexible and tunable gel systems.

In terms of applications, **DPP-1** was incorporated as an additive in OSCs. The presence of the **DPP-1** gel in the active layer significantly enhances the power conversion efficiency (PCE), increasing it from 6.37% to 7.85%. This improvement is attributed to the enhanced nanophase separation within the active layer, which facilitates more efficient charge transport and energy transfer in the solar cell. The optical properties of **DPP-1**, such as its absorption at 550 nm, complement the absorption spectrum of the active layer (PTB7:PC71BM), thus improving the external quantum efficiency (EQE) of the solar cell. Additionally, the nanorod network formed by the **DPP-1** gel in the active layer reduces the series resistance and enhances the fill factor of the devices.

The article presents **DPP-1** as an innovative gelator for 1D supramolecular assemblies, demonstrating its potential to enhance organic electronic devices such as solar cells. The ability of **DPP-1** to form J-type aggregates in both solid and gel states, along with its tunable optical and electronic properties, positions it as a promising material for future applications in organic electronics and smart devices [62].

**Figure 5:**
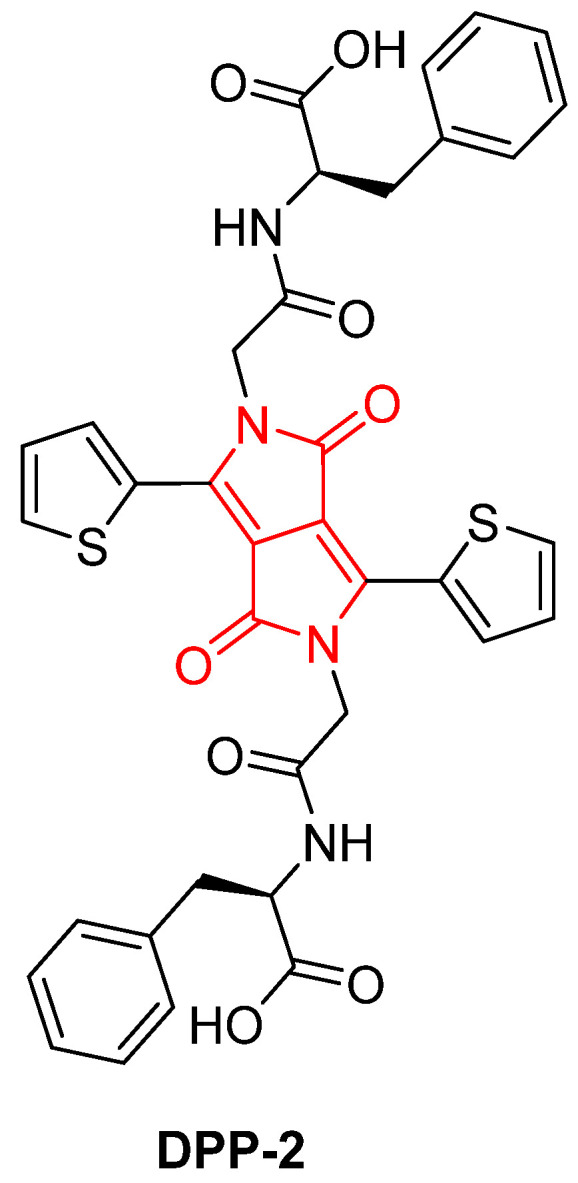
Chemical structure of **DPP-2**, described by Draper, Dietrich, and Adams (2017) [63].

In 2017, Draper, Dietrich, and Adams introduced the first example of a DPP-based hydrogelator capable of self-assembly in water, triggered by pH changes. The gelator, **DPP-2** (Figure 5), forms aggregated entangled structures at low pH and worm-like micelles at high pH. At high pH, these structures can be aligned using shear forces to create conductive materials. The study explores the gelation behavior, self-assembly, self-sorting, and electronic properties of this DPP-based system, highlighting its potential applications in organic electronics [63].

**Figure 6:**
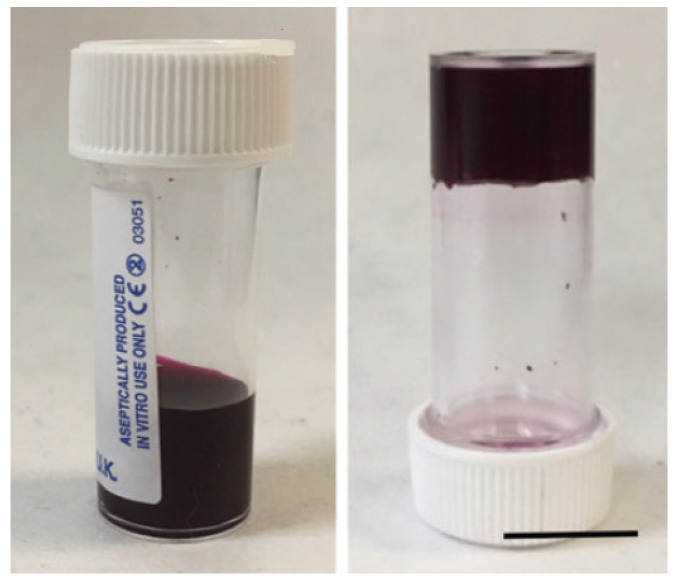
Gelation process of **DPP-2**, described by Draper, Dietrich, and Adams (2017) [63]. Image taken from reference [63].

The **DPP-2** gelator was synthesized through a series of reactions, including condensation, functionalization with phenylalanine, and peptide coupling. These modifications enabled the molecule to be solubilized at high pH and to drive self-assembly upon protonation. Gelation was achieved by gradually lowering the pH of the solution using glucono-d-lactone (GdL), resulting in the formation of a homogeneous hydrogel at pH 3.3 (Figure 6). Rheological measurements and scanning electron microscopy (SEM) were employed to confirm the gel’s structure, revealing a fibrous network characteristic of low molecular weight gelators (LMWGs). The gel exhibited a yield stress of approximately 900 Pa, indicating its high mechanical strength.

The article also investigates the electronic properties of **DPP-2** by measuring the resistivity of both dried xerogels and solutions. The xerogels exhibited improved conductivity, particularly after exposure to iodine vapor, which reduced their resistivity. This suggests that **DPP-2** xerogels may be suitable for use in organic electronic devices, such as organic field-effect transistors (OFETs), showing promise for electron donor applications in organic p–n heterojunctions.

Additionally, the authors demonstrated the self-sorting behavior of **DPP-2** when co-assembled with a perylene-based n-type gelator (PBI-A). The system displayed a slight shift in the wavelength response toward the visible range; however, the current produced was lower compared to PBI-A alone. This reduction in current was likely due to inefficiencies in the p–n heterojunction, which could have resulted from poor alignment and charge recombination between the two gelators. Nevertheless, the ability to align **DPP-2** structures under shear forces was shown to enhance their conductivity, as the aligned structures facilitated more efficient charge transport [63].

**Figure 7:**
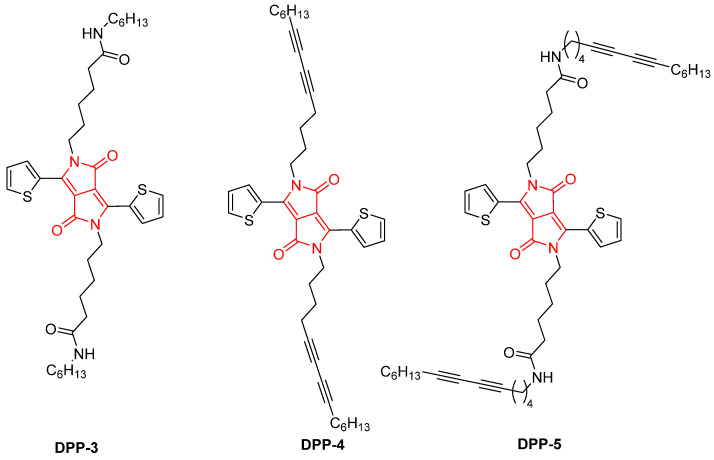
Chemical structure of **DPP-3**, **DPP-4,** and **DPP-5** reported by Nyayachavadi, Mason, Tahir, Ocheje, and Rondeau-Gagné (2018) [64].

In 2018, Nyayachavadi, Mason, Tahir, Ocheje, and Rondeau-Gagné reported a novel approach to cross-linking DPP-based organogels using polydiacetylenes (PDAs) through topochemical polymerization. In their study, the authors developed new DPP-based gelators (**DPP-3**, **DPP-4**, and **DPP-5**) by incorporating amide moieties into their side chains, promoting the formation of intermolecular hydrogen bonds that facilitate gelation (Figure 7) [64]. These gels were able to self-assemble into dense fibrous networks when dissolved in aromatic solvents. Upon UV irradiation, the diacetylene groups in the gelators underwent photopolymerization, resulting in cross-linked materials with enhanced stability.

**Figure 8:**
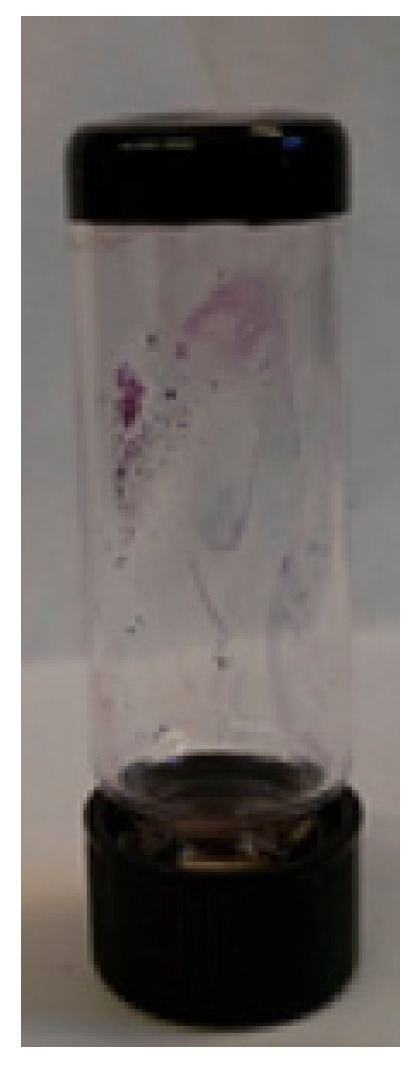
Gel obtained by **DPP-5** [64]. Image taken from reference [64]. Reprinted (adapted) with permission from (Langmuir **2018**, 34, 12126–12136). Copyright (2025). American Chemical Society.

**Figure 9:**
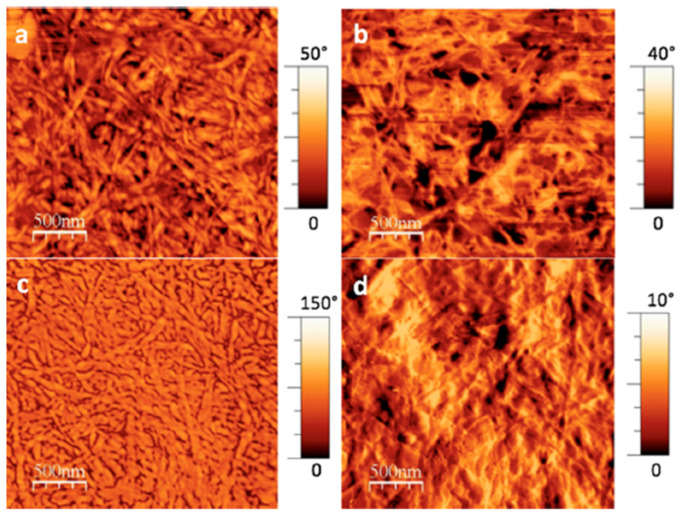
AFM images of organogels derived from **DPP-5** in different solvents such as (**a**) o-xylene, (**b**) benzene, (**c**) chlorobenzene, and (**d**) toluene. Image taken from reference [64]. Reprinted (adapted) with permission from (Langmuir **2018**, 34, 12126–12136). Copyright (2025). American Chemical Society.

The three gelators were synthesized through a straightforward alkylation reaction, which introduced diacetylene moieties to the DPP core via a process optimized for efficient gel formation. Among these gelators, **DPP-5**, which incorporated both amide and diacetylene groups, exhibited the most favorable gelation properties in solvents like toluene, benzene, o-xylene, or chlorobenzene (Figure 8). The resulting gels were characterized using several techniques, including AFM (Figure 9), X-ray diffraction (XRD), and Raman spectroscopy. Upon UV exposure, **DPP-5** successfully underwent cross-linking to form PDA. This cross-linking process significantly enhanced the electronic and optical properties of the material, rendering it suitable for applications in organic electronics.

The study also emphasizes the crucial role of intermolecular hydrogen bonding in facilitating the alignment of diacetylene moieties, which is essential for the successful topochemical polymerization. Furthermore, the researchers demonstrated that this approach enables the creation of well-defined nanomaterials without the need for external catalysts or additives, thereby opening new avenues for designing functional π-conjugated materials with controlled structures and properties.

By investigating the photopolymerization process and the structural integrity of the gels after irradiation, the study concludes that DPP-based gelators, such as **DPP-5**, can be employed to create stable, electroactive materials with potential applications in organic photovoltaics and electronics. This methodology also offers a versatile strategy for producing cross-linked materials with enhanced properties, paving the way for future advancements in electronic and optoelectronic applications [64].

**Figure 10:**
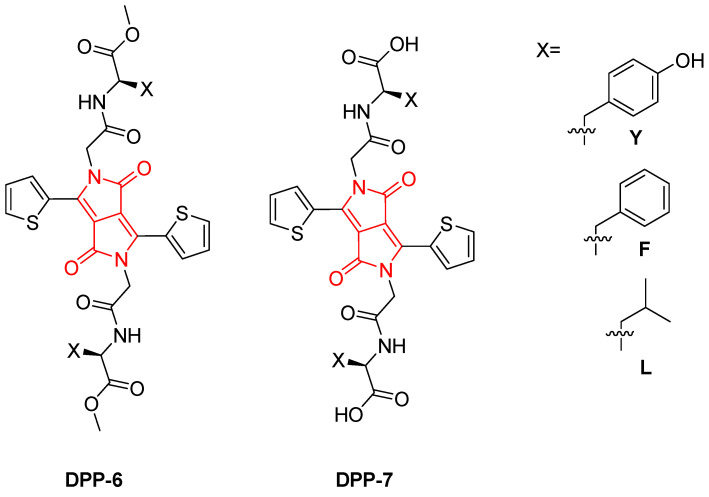
Chemical structure of family of **DPP-6** and **DPP-7** derivatives with phenylalanine (F), tyrosine (Y), and leucine (L) [65].

In 2020, Biswas, Sankarsan, et al. explored the development of supramolecular photocatalytic hydrogels based on **DPP-6** and **DPP-7** derivatives (Figure 10). The DPP core, functionalized with amino acid methyl esters such as phenylalanine (F), tyrosine (Y), and leucine (L), served a dual role in these systems as a structural component for self-assembly into hydrophobic fibers within the hydrogel and as a catalytic site for photochemical reactions [65].

The study highlights the ability of the DPP chromophore to generate singlet oxygen (^1^O_2_) upon visible light irradiation. This reactive oxygen species facilitates the selective oxidation of aromatic (thioanisole) and aliphatic (cyclohexyl methyl sulfide) substrates in aqueous environments. The enzymatic hydrolysis of the DPP-amino acid precursors leads to a rebalancing of amphiphilicity, enabling the formation of unidirectional supramolecular assemblies stabilized by π–π interactions, hydrogen bonding, and hydrophobic effects.

**Figure 11:**
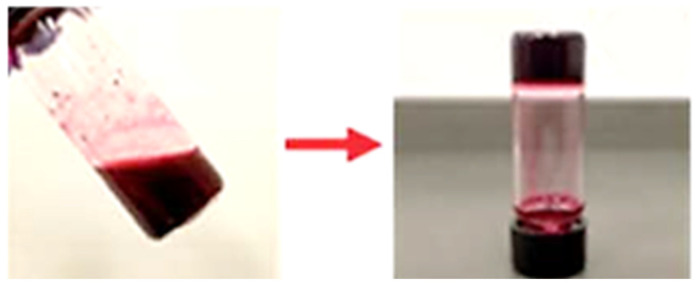
Gels obtained from **DPP-6Y** [65]. Image taken from reference [65]. Reprinted (adapted) with permission from (Chem. Sci. **2020**, 11, 4239–4245). Copyright (2025). Royal Society of Chemistry.

The research underscores the impact of amino acid side chains on the properties of the hydrogels, influencing their aggregation state (H-/J-mixed aggregates), gelation capability, and photocatalytic efficiency. For example, **DPP-6Y** and **DPP-6F** derivatives formed robust hydrogels (Figure 11) with efficient ^1^O_2_ production, achieving yields as high as 100% in the oxidation of cyclohexyl methyl sulfide. In contrast, the leucine derivative, **DPP-6L**, which did not form a hydrogel, exhibited significantly lower catalytic performance, thereby emphasizing the crucial role of hydrogelation in enhancing substrate accessibility and stabilizing reactive intermediates.

Moreover, the study demonstrates that using deuterium oxide (D_2_O) as a solvent significantly enhances catalytic yields due to the extended lifetime of ^1^O_2_ in this medium. This approach effectively prevents overoxidation to undesired sulfone products, achieving high chemoselectivity. The results suggest that these DPP-based hydrogels offer a sustainable, metal-free platform for photocatalytic applications, including organic synthesis, water treatment, and potentially photodynamic therapy.

The work highlights the versatility of DPP as a chromophore for creating hierarchical structures with emergent photophysical properties, paving the way for innovative catalytic systems under environmentally friendly conditions [65].

**Figure 12:**
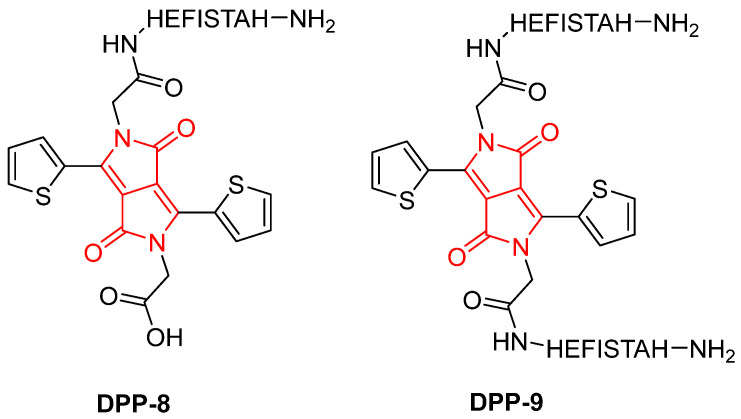
Chemical structure of **DPP-8** and **DPP-9** described by Aakanksha Rani et al. [66].

In the same year (2020), Aakanksha Rani et al. investigated the role of thiophene-diketopyrrolopyrrole (TDPP) as a critical component for gel formation in peptide-based bio-organic materials. By conjugating TDPP to the octapeptide HEFISTAH, the authors achieved enhanced self-assembly and hydrogelation, leading to the formation of gels. Two systems were synthesized, a mono-linked peptide (**DPP-8**) and a cross-linked peptide dimer (**DPP-9**), both of which were capable of forming hydrogels at a 4 wt% concentration under neutral pH conditions (Figure 12) [66].

**Figure 13:**
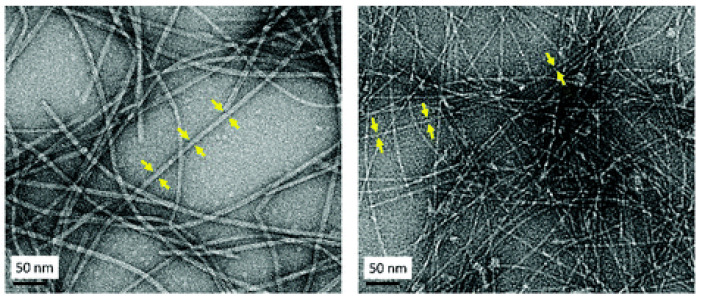
TEM image of bundles of fibers of **DPP-8** (**left**) showing obtained from a sample prepared at 1 wt% in water at pH = 7 and fibers of **DPP-9** (**right**) obtained from a sample prepared at 1 wt% in water at pH = 7. The yellow arrows indicate thickness of each fiber. Image taken from reference [66].

**Figure 14:**
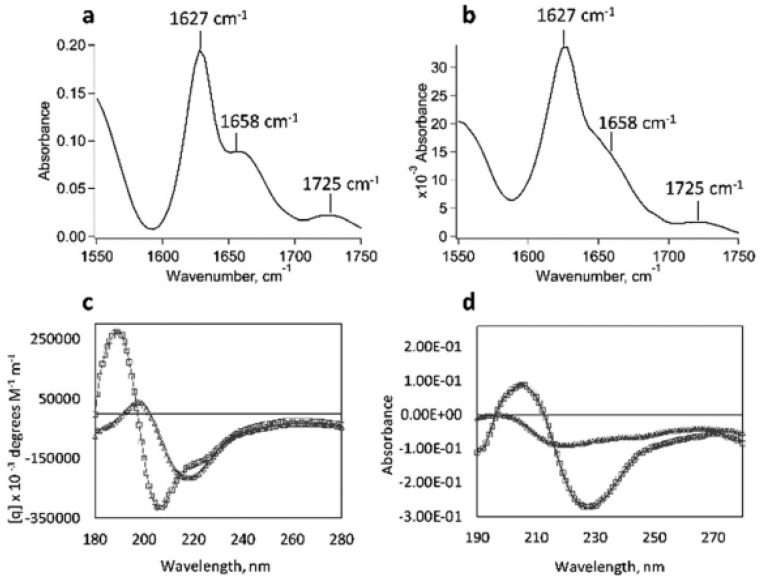
FTIR spectra of (**a**) **DPP-8** and (**b**) **DPP-9** at 4 wt% in water at pH = 7. (**c**) Circular dichroism spectra of solutions of **DPP-8** (open squares) and **DPP-9** (open triangles) at 0.5 wt%, 25 °C, and pH = 7 and (**d**) circular dichroism spectra of dried thin-films of **DPP-8** (open squares) and **DPP-9** (open triangles) at 25 °C prepared from a 1 wt% solution at pH = 7. Image taken from reference [66].

**Figure 15:**
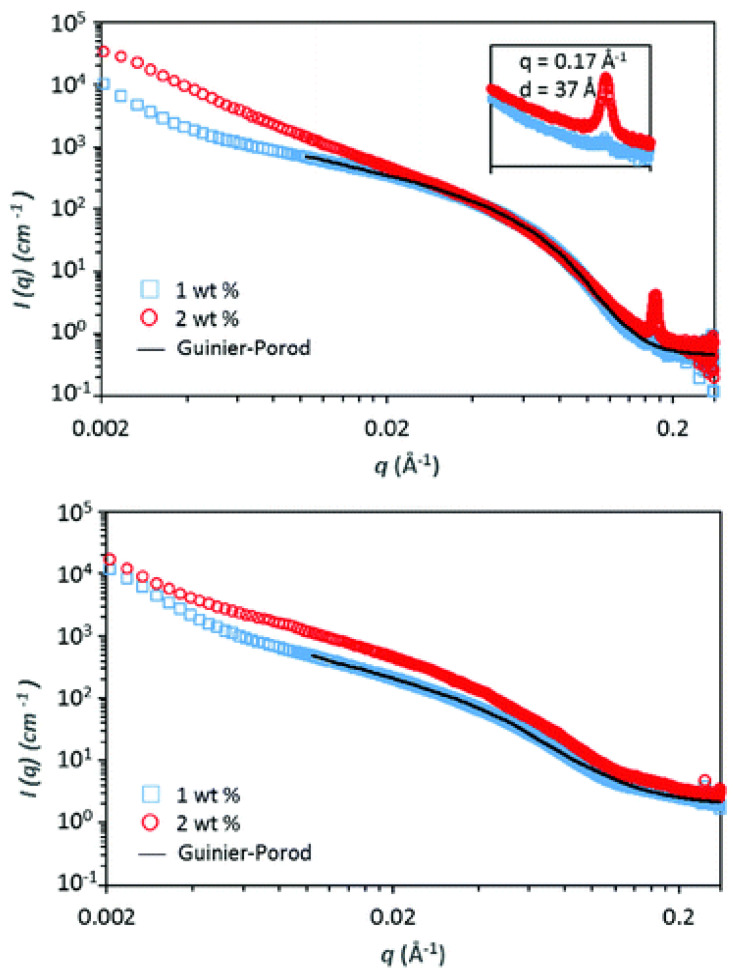
SAXS profiles of **DPP-8** (**top**) and **DPP-9** (**bottom**) in water at pH = 7 and at different concentrations. The inset figure in the top figure reflects the Bragg diffraction centered at 0.17 Å. Image taken from reference [66].

The TDPP core plays a central role in promoting gel formation by inducing π–π stacking interactions and facilitating hierarchical assembly into fibrous structures. This gelation capability is absent in the unmodified peptide, emphasizing the contribution of TDPP to the hydrogel’s stability and organization. Structural characterization through TEM (Figure 13), SAXS (Figure 14), and ATR-FTIR (Figure 15) confirmed that these hydrogels adopt β-sheet secondary structures with ordered molecular arrangements.

The hydrogels exhibit remarkable mechanical properties, with the mono-linked peptide (**DPP-8**) forming significantly stiffer gels than the cross-linked dimer (**DPP-9**). Furthermore, the TDPP chromophores within the gel promote H-aggregation, enhancing charge transport potential. These characteristics were retained in thin films derived from the hydrogels, demonstrating that the structural integrity and electronic properties of the gels are effectively translated into solid-state materials.

The work underscores the dual role of TDPP as both a gelation-inducing agent and an optoelectronic enhancer. By enabling the formation of stable hydrogels with tunable properties, TDPP–peptide conjugates pave the way for applications in biosensing, bioelectronics, and the fabrication of bio-organic thin films [66].

**Figure 16:**
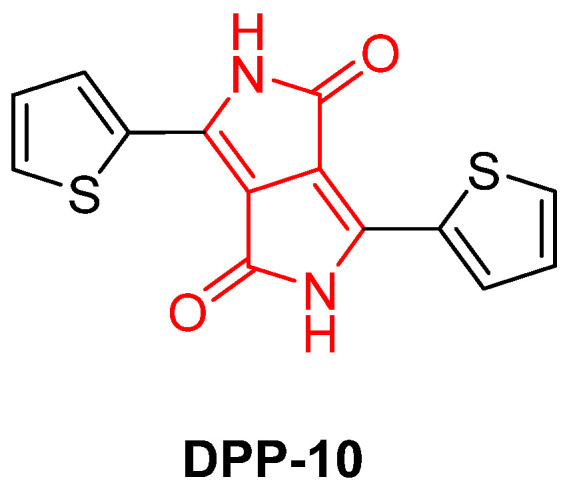
Chemical structure of **DPP-10** described by Kumar et al. [67].

In 2021, Kumar et al. explored the role of DPP (**DPP-10**) as an additive in poly(ethylene oxide) (PEO)-based gel polymer electrolytes (GPEs) to improve the performance of dye-sensitized solar cells (DSSCs) (Figure 16). The study demonstrates that the incorporation of **DPP-10** significantly enhances the ionic conductivity and diffusion coefficient of the PEO-based electrolytes, leading to improved photovoltaic performance [67].

**Figure 17:**
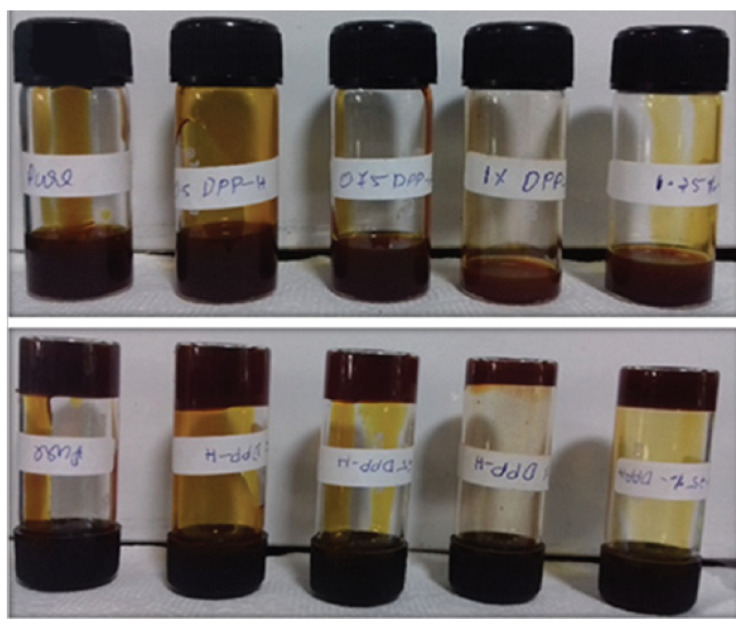
Different photographs of the pure and **DPP-10**-doped gel polymer electrolytes [67]. Image taken from reference [67]. Reprinted (adapted) with permission from (Dalton Trans. **2021**, 50, 7647–7655). Copyright (2025). Royal Society of Chemistry.

The addition of **DPP-10** to the PEO/propylene carbonate (PC) electrolyte reduces the crystallinity of the polymer, making it more amorphous. This structural change is crucial as it enhances ionic transport within the gel, facilitating the movement of iodine ions and thereby improving the electrolyte’s conductivity. The optimized incorporation of **DPP-10** (0.75 wt%) resulted in a maximum ionic conductivity of 0.393 mS cm^−1^, a substantial increase compared to the pure PEO/PC electrolyte (Figure 17). This enhancement in conductivity is attributed to **DPP-10**′s ability to disrupt the crystalline structure of PEO, which increases chain flexibility and promotes faster ionic movement.

The gel polymer electrolytes (GPEs) with **DPP-10** were characterized using X-ray diffraction (XRD) and Fourier transform infrared spectroscopy (FTIR), confirming a reduction in crystallinity and the formation of a more amorphous structure. The presence of **DPP-10** also facilitates the formation of a charge-transfer complex with iodine, further enhancing the ionic conductivity and improving the electrochemical properties of the gel.

In DSSC applications, the optimized **DPP-10**-modified GPE (PD-0.75) demonstrated a PCE of 6.69%, compared to 4.39% for the pure electrolyte. The incorporation of **DPP-10** improved photovoltaic performance by enhancing ion diffusion and reducing interfacial resistance. Electrochemical impedance spectroscopy (EIS) and Bode plot analysis revealed that the **DPP-10**-modified GPE exhibited lower charge transfer resistance and longer electron relaxation lifetime, contributing to the enhanced efficiency of the DSSCs.

The work highlights the dual role of **DPP-10** as both a gelation agent and an enhancer of ionic conductivity in gel polymer electrolytes. The study suggests that **DPP-10** could serve as a promising candidate for developing high-performance gel electrolytes in DSSC applications, offering a more stable and efficient alternative to traditional liquid electrolytes [67].

**Figure 18:**
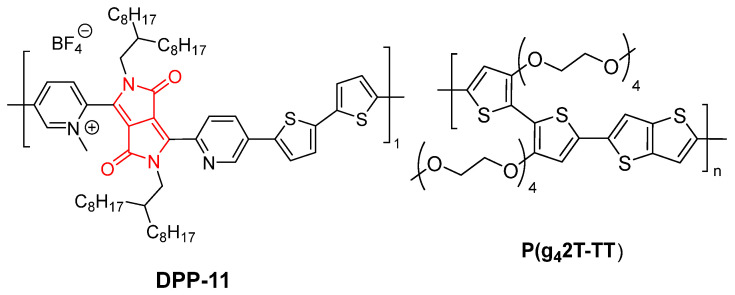
Chemical structure of **DPP-11** and the co-polymer P(g42T-TT) described by Stegerer et al. [68].

In 2022, Stegerer et al. described how DPP-based copolymers can form organogels through blending with other polymers, showcasing novel optical and electronic properties. In their study, an n-type copolymer based on DPP, named **DPP-11**, was combined with a p-type polymer, P(g42T-TT), resulting in organogels that exhibit solid-state electron transfer (Figure 18). This behavior is unique to this combination due to the specific molecular interactions between the polymers [68].

**Figure 19:**
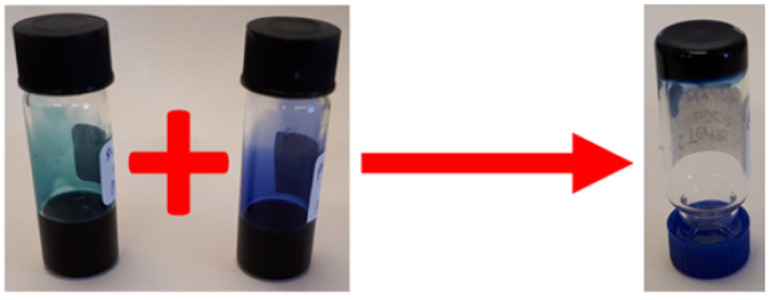
**DPP-11**:P(g42T-TT) organogel formation in o-dichlorobenzene [68]. Image taken from reference [68]. Reprinted (adapted) with permission from (Macromolecules **2022**, 55, 4979–4994). Copyright (2025). American Chemical Society.

The role of DPP in gel formation is critical. First, the chemical modification of DPP through methylation (resulting in **DPP-11**) significantly reduces the material’s Lowest Unoccupied Molecular Orbital (LUMO) level, enhancing its electronic compatibility with P(g42T-TT). This compatibility is essential for facilitating solid-state electron transfer, a key factor for the electronic applications investigated. Second, the blending of **DPP-11** with P(g42T-TT) in dichlorobenzene solutions leads to the rapid formation of organogels with thixotropic properties, suggesting a unique interaction between these molecular structures (Figure 19). This phenomenon is not observed with other DPP copolymers, highlighting the distinctive role of the modified DPP in this gelation process.

Furthermore, DPP’s ability to form these gels offers practical advantages, as it allows for the simultaneous processing of both p-type and n-type polymers in homogeneous solutions. This simplifies the fabrication of multilayer films and electronic devices, ensuring the proper orientation and distribution of components. In conclusion, DPP derivatives are not only crucial for their contribution to electron transfer but also for their ability to generate unique gel structures through specific molecular interactions, positioning them as key materials in advanced organic electronic applications [68].

**Figure 20:**
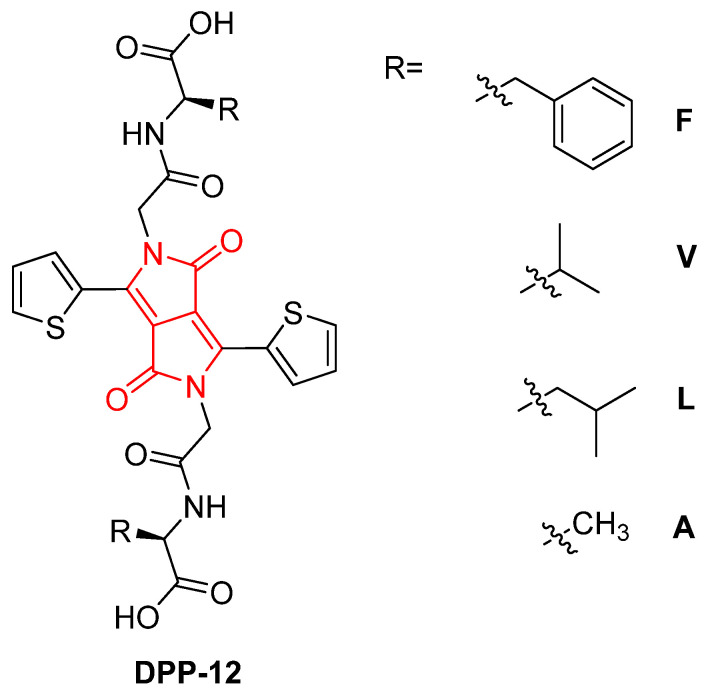
Chemical structure of **DPP-12V** described by Gauci et al. [69].

In a related study in 2022, similar to the previously reported in reference [65], Gauci et al. explored the synthesis and characterization of hydrogels derived from DPP derivatives functionalized with amino acids, which undergo a pH-triggered gelation process [69]. The research expands the scope of DPP-based supramolecular gels and demonstrates how molecular modifications can influence gelation behavior, self-assembly, and mechanical properties. The core of these derivatives, dithiophene-DPP (DTDPP), promotes π–π stacking and hydrophobic interactions, essential for the formation of fibrous networks and gels. Further functionalization with amino acids, such as phenylalanine, valine, leucine, and alanine, introduces hydrogen bonding and other intermolecular interactions that drive self-assembly, leading to the formation of different **DPP-12** derivatives (Figure 20).

**Figure 21:**
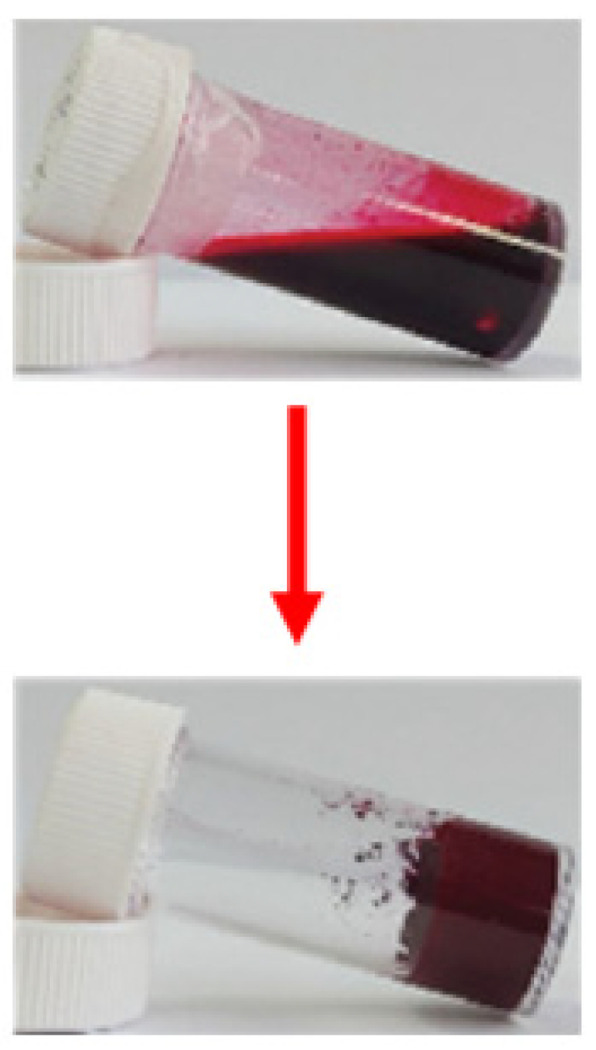
Example of gels at pH = 10.5 obtained from **DPP-12V** described by Gauci et al. [69]. Image taken from reference [69].

In an aqueous environment at high pH (pH 10.5), the **DPP-12** derivatives dissolve due to the deprotonation of their carboxylic acid groups. Upon the gradual addition of glucono-d-lactone (GdL), the pH decreases, leading to the re-protonation of the carboxylic acid groups and the subsequent formation of fibrous structures that eventually evolve into hydrogels (Figure 21). This transition is marked by a shift from micellar aggregates to a three-dimensional gel network. The study reveals that the mechanical properties of the hydrogels vary significantly depending on the type of amino acid functionalization. For example, aromatic amino acids like phenylalanine produce stiffer gels due to enhanced π-interactions, while aliphatic residues such as valine and leucine result in weaker gels. These differences are also reflected in the minimum gelation concentration, rheological behavior, and pKa values of the derivatives.

**Figure 22:**
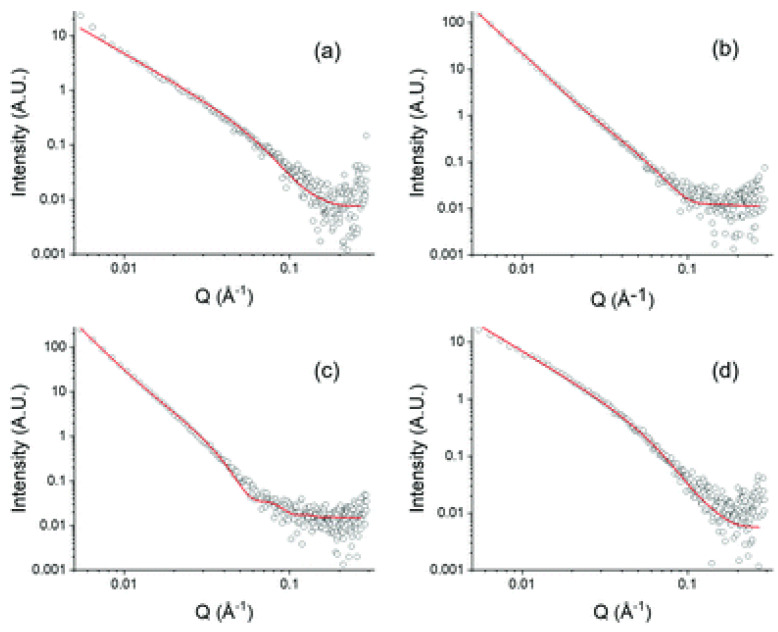
SAXS data for **DPP-12** gels (open black circles) with form factor fits (red line) for (**a**) **DPP-12F**; (**b**) **DPP-12V**; (**c**) **DPP-12L**; (**d**) **DPP-12A**. Image taken from reference [69].

**Figure 23:**
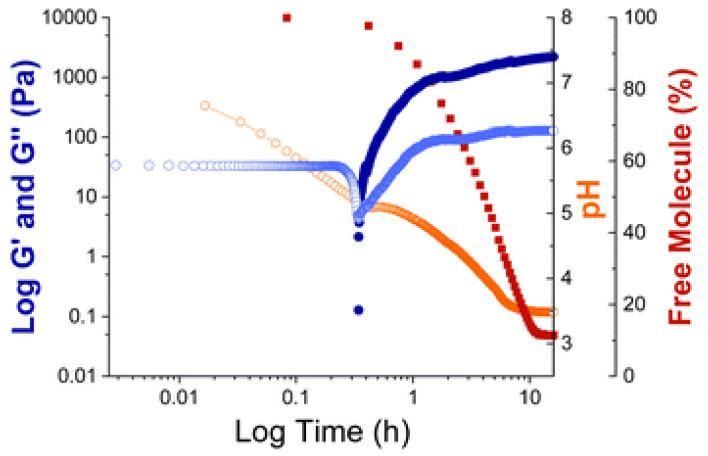
Rheology experiments with time sweep analysis for **DPP-12A** with G′ (dark blue), G″ (light blue), pH (orange), and % free molecule by NMR spectroscopy (dark red) shown with time as the gelation takes place after addition of GdL. Image taken from reference [69].

The study’s comprehensive analysis includes rheological measurements, small-angle X-ray scattering (SAXS), and NMR spectroscopy, which confirm the formation of anisotropic fibrous structures that form the gel network. SAXS data reveal that the dimensions and crosslinking density of these fibers vary depending on the functional group (Figure 22), while rheological tests demonstrate the gels’ mechanical stability under different conditions (Figure 23). Notably, the **DPP-12** derivatives exhibit shear-thinning behavior, thermal stability up to 80 °C, and partial recovery after high strain.

The research emphasizes the potential of DPP-based hydrogels for advanced applications, such as photocatalysis, where the self-assembled structure can influence material performance. The tunability of gel properties through functionalization and preparation conditions offers opportunities to design materials with customized mechanical and chemical characteristics. By bridging molecular design with material functionality, the study highlights the versatility and promise of DPP derivatives in supramolecular gel chemistry and functional materials [69].

In summary, these examples of the current state of the art in the application of DPP in gels highlight the significant potential of this moiety for such applications and underscore the largely unexplored opportunities offered by this well-established organic compound in this field.

## 3. Future Perspectives

The diketopyrrolopyrrole (DPP) moiety represents a highly promising candidate in the field of gel-forming materials, because of its unique features. These include its capacity for self-assembly, remarkable thermal and optical stability, and a pronounced tendency to engage in π–π interactions. However, the application of DPP in gel-based systems is still in its early stages. This early phase of exploration offers significant opportunities to further investigate the potential of these systems and their diverse applications across various scientific and technological domains.

1. Optimization of Molecular Architecture: The strategic design of DPP derivatives for gel formation holds considerable promise for advancing material science. By modifying the side chains attached to the DPP core, it is possible to induce and enhance self-assembly mechanisms and significantly improve the stability of the resulting gels. Incorporating functional groups that promote hydrogen bonding, electrostatic interactions, or hydrophobic forces enables precise control over the mechanical and thermal properties of the gels. Additionally, the integration of DPP with other functional units makes possible the development of hybrid gels, which could exhibit advanced and multifunctional properties tailored for specific applications.

2. Development of Stimuli-Responsive Gels: A promising direction for future research involves the design of DPP-based gels capable of responding to external stimuli, such as temperature, light, pH, or electric fields. The intrinsic photoconductive and light-absorbing properties of DPP makes it an ideal candidate for creating photosensitive gels, which could find applications in optical devices and work as actuators for soft robotics. Furthermore, incorporating DPP into systems that can react to multiple stimuli simultaneously could enable the creation of advanced sensors and intelligent materials with a wide range of functionalities.

3. Innovations in Soft Materials and Flexible Electronics: The combination of DPP’s optical and electronic properties, along with the inherent adaptability of gels, makes it a highly promising material for applications in flexible electronics. By developing conductive or semiconductive gels that incorporate DPP, researchers could create innovative solutions for wearable devices, touch-sensitive interfaces, and electronic membranes. To advance this field, it will be crucial to gain a deeper understanding of how to maintain DPP’s electronic performance within gel matrices while ensuring the overall functionality and durability of the material.

4. Scalability and Sustainability: While DPP exhibits remarkable properties, significant challenges remain in scaling up its production and reducing costs. Future research efforts should concentrate on creating more economic, efficient, and sustainable synthetic routes for DPP derivatives, employing available raw materials and environmentally benign processes. Equally important is the need to explore the recyclability and biodegradability of DPP-based gels, as these factors will play a crucial role in positioning them as viable and eco-friendly alternatives to traditional synthetic materials.

5. Cross-Disciplinary Collaboration: Advancing the use of DPP as a gel-forming agent will require collaboration across multiple fields, including chemistry, physics, and engineering. Computational modeling can also play a key role in understanding molecular interactions within DPP gels, helping to identify and design optimized structures more efficiently.

6. Discovery of Novel Applications: Exploring new frontiers for DPP-based gels opens up exciting opportunities for future research. These materials could find use in innovative areas such as energy storage and harvesting, environmental engineering, and 3D printing technologies. For example, DPP gels can be implemented as electrolytes in flexible batteries or as absorbent materials in water purification systems. The inherent versatility of the DPP core suggests that its potential applications are far from being fully explored, pointing to countless possibilities for innovative and transformative uses.

## 4. Conclusions

This review examines the growing potential of diketopyrrolopyrrole (DPP) as a promising material in gels, an area that remains unexplored compared to the well-established roles in organic electronics and photonics. Analyzing existing literature, we have identified the structural and functional characteristics of DPP, positioning it as a high-potential candidate for gel systems. Its π–π stacking abilities, thermal stability, and versatile options for functionalization provide a robust foundation for developing gels with tailored properties.

It is worth noting that the few reports on DPP-based gels in the literature have emerged in the past decade, highlighting the novelty and increasing interest in this area. The studies demonstrate that DPP-based gels are capable of self-assembly under various conditions, forming structures with diverse applications in soft materials and molecular engineering. The strategic molecular design of derivatives, particularly through the incorporation of different functional substituents, plays a crucial role in modulating gelation behavior and optimizing the final performance.

Despite the promising developments, the field remains in its early stages. The limited number of studies underscores the need for more comprehensive exploration to fully exploit the potential of DPP-based gels. Challenges such as scalable synthesis, the design of novel functional derivatives, and the integration of DPP gels into practical applications indicate the future research directions in this field.

In conclusion, this review highlights the potential of the DPP core in gel field. The notable increase in interest over the past decade suggests that DPP-based gels may become a cornerstone in the design of next-generation materials, bridging the gap between molecular functionality and macroscopic performance.

## Data Availability

The data are contained within this article.
